# Chaperone gp96 mediates ER-α36 cell membrane expression

**DOI:** 10.18632/oncotarget.5273

**Published:** 2015-09-10

**Authors:** Junwei Hou, Mengmeng Deng, Xin Li, Weiwei Liu, Xiaoyu Chu, Jing Wang, Feng Chen, Songdong Meng

**Affiliations:** ^1^ CAS Key Laboratory of Pathogenic Microbiology and Immunology, Institute of Microbiology, Chinese Academy of Sciences (CAS), Beijing 100101, P.R. China; ^2^ Shenogen Pharma Group, Changping District, Beijing 102206, P.R. China

**Keywords:** ER-α36, gp96, ubiquitin, MAPK, breast cancer

## Abstract

ER (estrogen receptor)-α36, a variant of human ERα, activates non-genomic cell signaling pathways. ER-α36 on the cell membrane plays a role in breast cancer growth and development, and contributes to tamoxifen resistance. However, it is not understood how cell membrane expression of ER-α36 is regulated. In this study, we investigated the role of cell membrane glycoprotein 96 (mgp96) in the regulation of ER-α36 expression and signaling. We found that the C-terminal domain of mgp96 directly interacts with ER-α36 on the cell membrane of breast tumor cells. This interaction stabilizes the ER-α36 protein, thereby increasing its signaling, which, in turn, increases tumor cell growth and invasion. Moreover, targeting mgp96 with siRNA or monoclonal antibody (mAb) blocks the mgp96-ER-α36 interaction and inhibits breast cancer growth and invasion both *in vitro* and *in vivo*. These results provide insights into the modulation of cell membrane ER-α36 expression and suggest that mgp96 could be a potential therapeutic target for ER-α36-overexpressing breast cancer.

## INTRODUCTION

Breast cancer accounts for 22.9% of all cancers in women [1]. It is a hormone-dependent disease, with estrogens playing a dominant role in both cancer initiation and progression. The biological activities of estrogens are mediated through estrogen receptors (ERs), which are expressed by nearly 70% of breast tumors. Thus, tamoxifen, an ER agonist, has been used to treat ER-positive breast cancer for over 30 years [2]. However, many patients with ER-positive tumors develop resistance to tamoxifen therapy, posing a challenge for treatment [3].

ER-α36, a variant of human ERα, is involved in tamoxifen resistance. Compared to full-length (66 kDa) ERα, ER-α36 lacks both transcriptional activation domains (AF-1 and AF-2) while retaining the DNA-binding domain and partial dimerization and ligand-binding domains [4]. Its C-terminal 27-amino acid domain is unique and takes the place of the last 138 amino acids encoded by exons 7 and 8 of the ESR1 gene. ER-α36 is mainly expressed in the cytoplasm, as well as on the cell surface where it mediates non-genomic estrogen and anti-estrogen signaling via intracellular signaling pathways (such as MAPK/ERK) and promotes cell growth [5].

ER-α36 signaling via MAPK/ERK and PI3K/AKT pathways promotes tamoxifen actions in endometrial cancer cells [6]. ER-α36 also increases Epidermal Growth Factor Receptor (EGFR) expression and decreases ERα expression, which could be an underlying mechanism for acquired tamoxifen resistance in breast cancer [7]. Moreover, approximately 40% of ERα-positive breast cancer patients have high levels of ER-α36 in their tumors, and this subset of patients are less likely to receive benefits from tamoxifen therapy compared to those with ERα-positive/ER-α36-negative tumors [5].

GRP94, also known as gp96, is an endoplasmic reticulum-resident member of the cytosolic heat shock protein 90 (HSP90) family. Gp96 is a molecular chaperone participating in glycoprotein folding and facilitating the degradation of misfolded proteins [8]. Interestingly, the endoplasmic reticulum-resident gp96 translocates to the cell membrane in certain tumor cells [9, 10]. Moreover, membrane expression of mgp96 is related to malignancy in breast cancer [11], and elevated gp96 correlates with tumor progression and ER-α36 expression in gastric cancer [12].

We previously found that mgp96 binds to human epidermal growth factor 2 (HER2) and EGFR, facilitating HER2 dimerization and signaling and promoting breast tumor growth [13]. ER-α36 also physically interacts with EGFR and HER2 and promotes malignant growth of breast cancer cells [14, 15]. Given the important roles of cell membrane ER-α36 in breast cancer growth and tamoxifen resistance, we investigated the role of mgp96 in the regulation of ER-α36 expression on the cell membrane. Our results offer a new therapeutic strategy for breast cancer treatment.

## RESULTS

### ER-α36 binds to gp96 on the cell membrane of breast cancer cells

**Figure 1:**
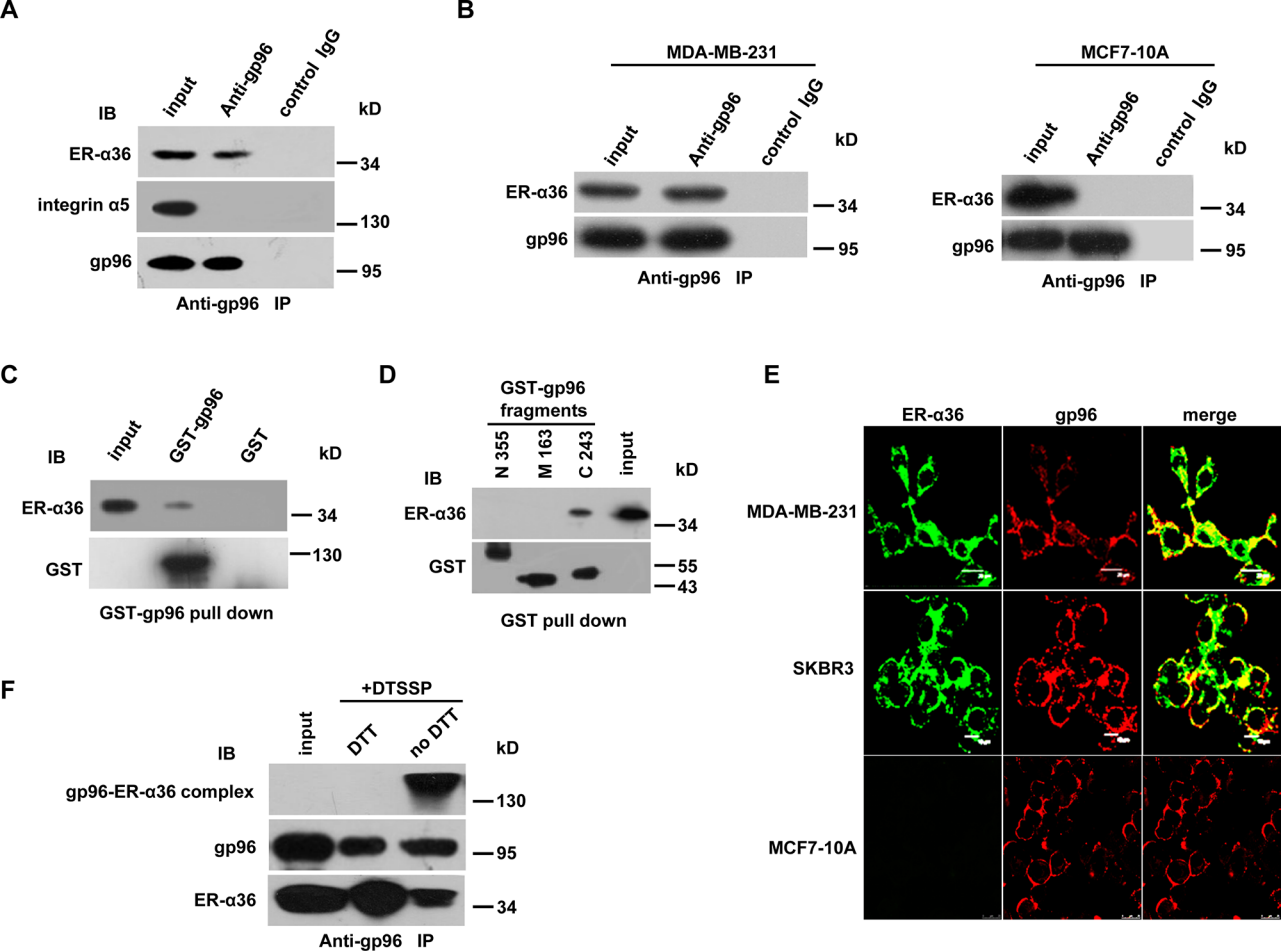
ER-α36 interacts with gp96 on the cell membrane of breast cancer cells **A.** and **B.** Co-IP assay with the anti-gp96 polyclonal antibody using total cell lysates (A) or cell membrane proteins (B) to test for the interaction between gp96 and ER-α36 in MDA-MB-231 and MCF7-10A cells. Cell membrane integrin α5 served as a negative control. **C.** and **D.** *In vitro* GST pull-down assays with purified GST-gp96 (C) or GST-gp96 fragments (N355, M163 and C243) (D) **E.** Detection of gp96 and ER-α36 by confocal microscopy in unpermeabilized SKBR3, MDA-MB-231, and MCF7-10A cells. Scale bar, 20 μm. **F.** Co-IP with the anti-gp96 polyclonal antibody in SKBR3 cells cross-linked with DTSSP. Cells were washed with cold PBS three times and cross-linked with membrane-nonpermeable, thiol-cleavable DTSSP (final concentration; 2 mM) on ice for 30 min. Cell lysates were immunoprecipitated with the anti-gp96 polyclonal antibody, and the immunoprecipitates were treated with or without DTT, and subjected to Western blot.

To determine whether gp96 interacts with ER-α36 in breast cancer cells, we performed a co-IP assay with anti-gp96 polyclonal antibody in ER-α36-positive MDA-MB-231 cells [14]. We found that gp96 interacts with ER-α36 in breast cancer cells (Figure 1A). Similar results were observed when the co-IP assay was performed with membrane proteins from MDA-MB-231 cells, but not with membrane proteins from ER-α36-negative MCF7-10A cells (Figure 1B), indicating the specificity of gp96 binding to ER-α36 on the cell surface. The interaction between gp96 and ER-α36 was further confirmed by GST pull-down assay (Figure 1C). To determine the region of gp96 involved in the gp96-ER-α36 interaction, we expressed a variety of truncated gp96 fragments (C243: aa 540–782, M163: aa 377–539, N355: aa 22–376). As shown in Figure 1D, the C-terminal domain of gp96, C243 (aa 540–782), interacted with ER-α36. Furthermore, confocal microscopy analysis showed that gp96 partly co-localized with ER-α36 on the cell membrane of ER-α36-positive MDA-MB-231 and SKBR3 cells but not on the cell membrane of ER-α36-negative MCF7-10A cells (Figure 1E). Next, cross-linking and co-IP with anti-gp96 polyclonal antibody was performed on the cell membrane of MDA-MB-231 cells. As shown in Figure 1F, gp96 associated with ER-α36 on the cell membrane.

### mgp96 positively regulates ER-α36 expression and enhances cell proliferation and invasion

**Figure 2:**
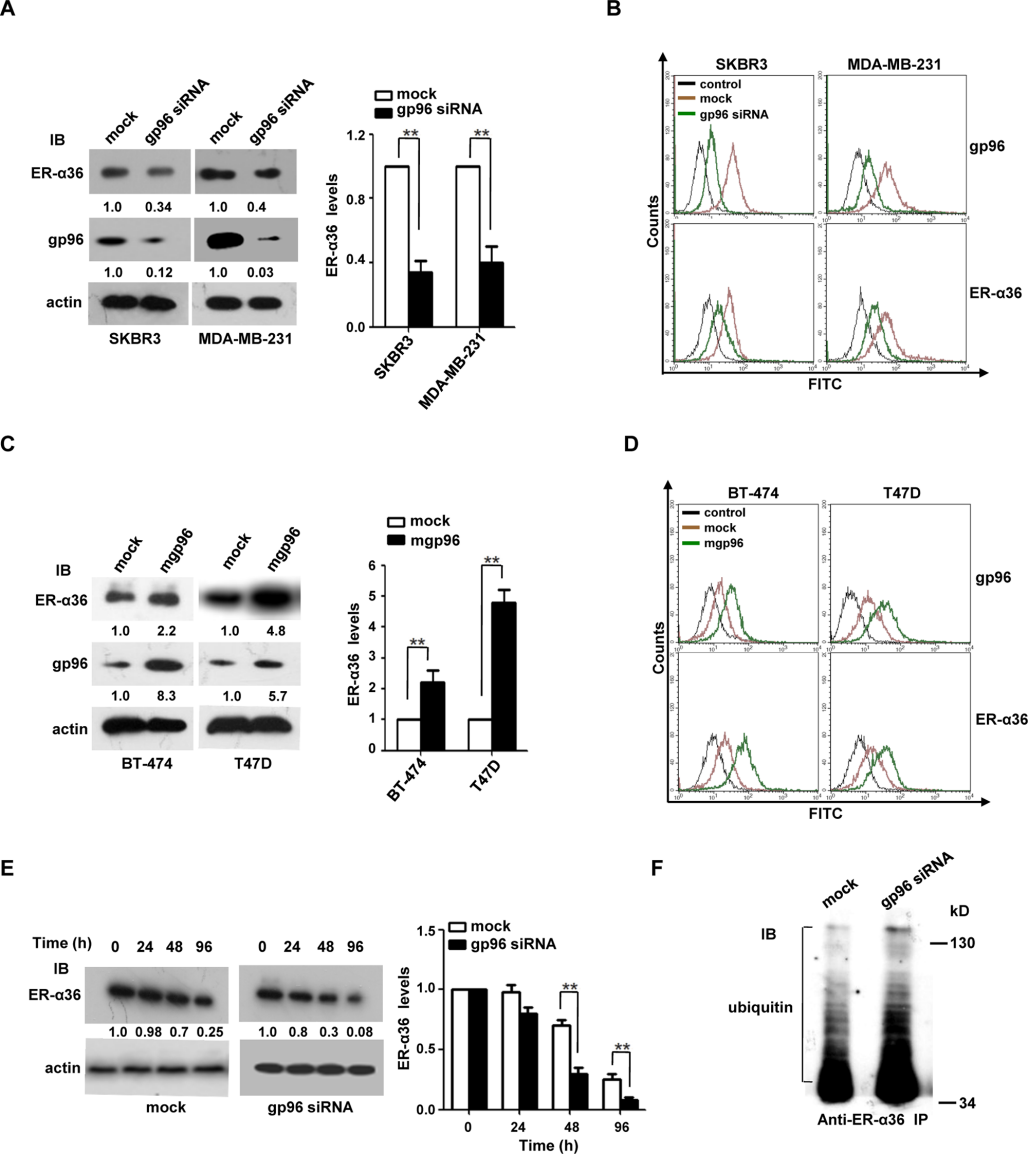
mgp96 upregulates the expression and stability of ER-α36 protein Breast cancer cells were pretreated with DMEM without phenol red (Hyclone, USA) and containing 2.5% fetal bovine serum (FBS) (Gibco, USA) for 48 h and maintained in the culture throughout the test. **A.** and **B.** SKBR3 and MDA-MB-231 cells were transfected with gp96 siRNA or control siRNA (mock) for 72 h. Total gp96 and ER-α36 levels were determined by Western blot and normalized by actin (A) Cell membrane gp96 and ER-α36 levels were detected by flow cytometry (B) Cells stained with control IgG served as a control. **C.** and **D.** BT-474 and T47D cells were infected with the adenoviruses ad-mgp96 or ad-pDC312 (mock) for 72 h. Total and cell membrane gp96 and ER-α36 levels were determined by Western blot (normalized by actin) (C) and flow cytometry (D), respectively. Cells stained with control IgG served as a control. **E.** The stability of ER-α36 protein was analyzed using a CHX chase experiment. MDA-MB-231 cells were transfected with gp96 siRNA or control siRNA (mock) for 36 h. Cells were then treated with 50 μg/ml CHX for the time as indicated, and cell lysates were subjected to Western blot. ER-α36 levels were normalized by actin. The ratio of ER-α36 to actin levels at 0 h was set as 1.0. **F.** Co-IP analysis of the ubiquitinated ER-α36 protein levels in MDA-MB-231 cells. Cells were transfected with gp96 siRNA or control siRNA (mock). Seventy-two hours after the transfection, cells were treated with 10 μM MG132 for 4 h. Cell lysates were immunoprecipitated with the anti-ER-α36 antibody, and immunoprecipitates were subjected to Western blot.

We next examined the effect of gp96 on ER-α36 expression. In our previous study, we found that gp96 was highly expressed on the membrane of MDA-MB-231 and SKBR3 cells and lowly expressed on BT-474 and T47D cells [13]. As shown in Figure 2A and 2B, gp96 knockdown significantly decreased both total and cell membrane ER-α36 levels. Compared to mock, depletion of gp96 decreased cell membrane ER-α36 in SKBR3 and MDA-MB-231 cells by 66.7% and 63.6%, respectively (both *P* < 0.01). Conversely, overexpression of mgp96 caused a dramatic increase in total (Figure 2C) and cell membrane (Figure 2D) ER-α36 levels. Overexpression of mgp96 increased cell membrane ER-α36 in BT-474 and T47D cells by ∼4-fold and ∼5-fold, respectively (both *P* < 0.01). However, there was no change in ER-α36 mRNA levels with gp96 knockdown or overexpression (data not shown), indicating that mgp96 does not regulate ER-α36 transcription.

Next we examined the impact of mgp96 on ER-α36 protein stability. Gp96 siRNA-treated cells showed a sharper time-dependent decrease in ER-α36 protein compared to mock-treated cells (Figure 2E), indicating that mgp96 affects ER-α36 protein stability. As ERα degradation mainly occurs via the ubiquitin-proteasome pathway [19–21], we quantified ER-α36 ubiquitination. As shown in Figure 2F, gp96 siRNA-treated cells had more ubiquitinated ER-α36 protein than mock-treated cells, suggesting that mgp96 regulates ER-α36 protein levels via the ubiquitin-proteasome degradation pathway.

**Figure 3:**
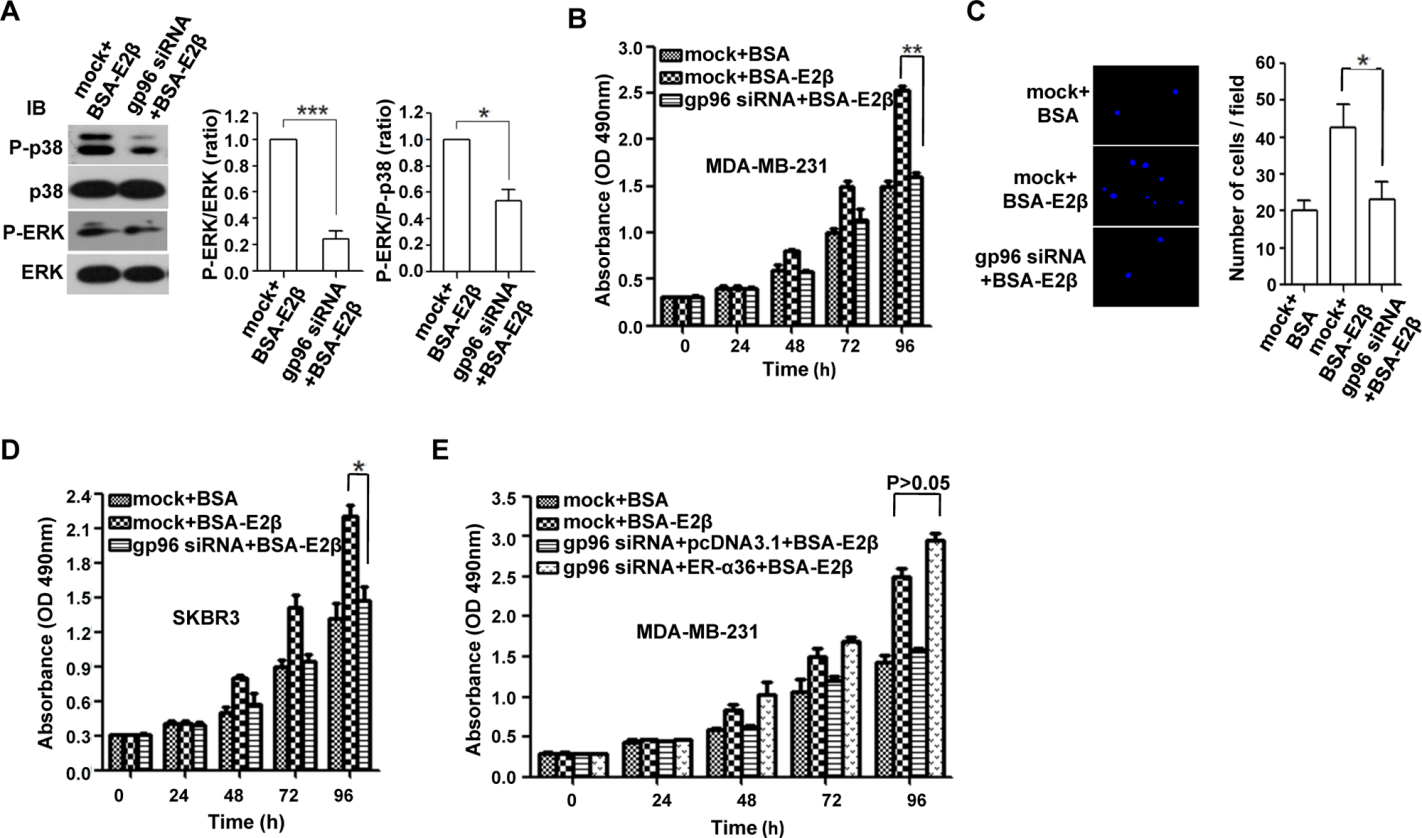
gp96 depletion reduces MAPK signaling and inhibits the growth and invasion of breast cancer cells Breast cancer cells were pretreated with DMEM without phenol red and containing 2.5% FBS for 48 h and maintained in the culture throughout the test. The stable shRNA cell lines MDA-MB-231-gp96i and MDA-MB-231-luci (mock) were treated with 50nM BSA-E2β for 20 min (A) 96 h (B) or 72 h (C). **A.** Western blot analysis of the protein levels of ERK, P-ERK, p38, and P-p38. Cell proliferation and invasion were analyzed by CCK-8 **B.** and transwell **C.** assays, respectively. **D.** SKBR3 cells transfected with gp96 siRNA or control siRNA (mock) were treated with 50nM BSA-E2β for 96 h, and cell proliferation was determined by CCK8 assay. **E.** MDA-MB-231-gp96i or MDA-MB-231-luci (mock) cells transfected with pcDNA-ER-α36 or empty vector pcDNA3.1 as control were treated with 50nM BSA-E2β for 96 h, and cell proliferation was determined by CCK8 assay. *P < 0.05, **P < 0.01, ***P < 0.001.

ER-α36 promotes breast tumor growth through the MAPK signaling pathway [22]. As shown in Figure 3A, gp96 knockdown decreased ERK phosphorylation (P-ERK) and led to a decreased ratio of P-ERK to P-p38. MDA-MB-231 cells with low HER2 expression were selected to determine the effect of targeting gp96 on cell proliferation and invasion, excluding the possibility that gp96 may affect cell growth via regulating HER2 dimerization [13]. As expected, gp96 depletion inhibited cell proliferation (Figure 3B) and invasion (Figure 3C) in both MDA-MB-231 cells and SKBR3 cells (Figure 3D). To further determine the effects of gp96 RNAi on cell growth via reduced ER-α36, an ER-α36 expression vector was transfected into the MDA-MB-231-gp96i cells. The result showed that inhibition of cell proliferation by gp96 knock-down was completely reversed by ER-α36 overexpression (Figure 3E).

### An anti-gp96 mAb blocks the mgp96-ER-α36 interaction

**Figure 4:**
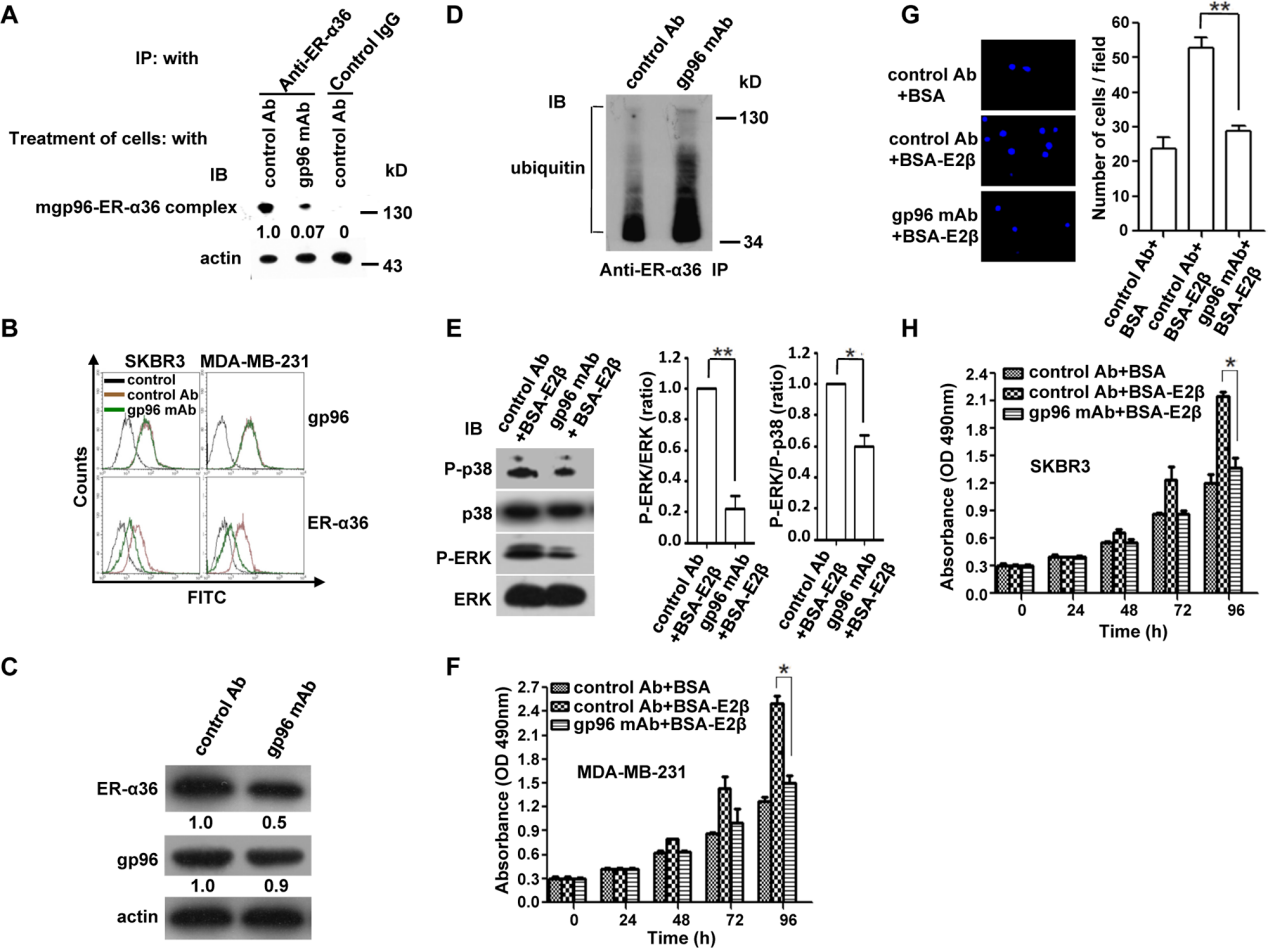
A gp96 mAb blocks the mgp96-ER-α36 interaction, decreases cell membrane ER-α36 levels, and suppresses growth and invasion of breast cancer cells SKBR3 and MDA-MB-231 cells were pretreated with DMEM without phenol red and containing 2.5% FBS for 48 h and maintained in the culture throughout the test. Cells were treated with the gp96 mAb or control antibody (50 μg/ml) for 8 h (A), 48 h (B–E), or 96 h (F and H), 72 h (G). **A.** Eight hours after the gp96 mAb treatment, MDA-MB-231 cells were cross-linked with the membrane-nonpermeable, thiol-noncleavable BS3 (final concentration; 2 mM) for 30 min on ice. Cell lysates were immunoprecipitated with the anti-ER-α36 monoclonal antibody, and the immunoprecipitates were subjected to Western blot. **B.** Cell membrane gp96 and ER-α36 abundance was analyzed by flow cytometry. Cells stained with control IgG served as a control. **C.** Western blotting analysis of total gp96 and ER-α36 protein levels in MDA-MB-231 cells. **D.** Co-IP analysis of the ubiquitinated ER-α36 levels in MDA-MB-231 cells. Cells treated with gp96 mAb were incubated with 10 μM MG132 for 4 h. Cell lysates were immunoprecipitated with the anti-ER-α36 antibody, and immunoprecipitates were subjected to Western blot. **E.** The protein levels of ERK, P-ERK, p38, and P-p38 were analyzed by Western blot in MDA-MB-231 cells co-treated with 50nM BSA-E2β for 20 min. **F.** Cell proliferation was analyzed by CCK-8 assay in MDA-MB-231 cells co-treated with 50nM BSA-E2β for 96 h. **G.** Cell invasion was determined by transwell assay in MDA-MB-231 cells co-treated with 50nM BSA-E2β for 72 h. **H.** Cell proliferation was analyzed by CCK-8 assay in SKBR3 cells co-treated with 50nM BSA-E2β for 96 h. *P < 0.05, **P < 0.01.

Multiple monoclonal antibodies against gp96 have been generated by our lab, and for this study we selected a gp96 mAb that efficiently blocks the activity of cell surface gp96 [18, 23]. Cross-linking and co-IP analyses revealed that the gp96 mAb blocked the association of ER-α36 with mgp96 (Figure 4A). Treatment of MDA-MB-231 and SKBR3 cells with the gp96 mAb reduced cell membrane ER-α36 levels (∼60% and ∼75%, respectively) (Figure 4B) and total ER-α36 protein levels (Figure 4C), and increased ER-α36 ubiquitination (Figure 4D). Treatment of MDA-MB-231 cells with the gp96 mAb also significantly inhibited ER-α36-mediated MAPK signaling (Figure 4E) and pronouncedly suppressed cell growth (Figure 4F) and invasion (Figure 4G). The inhibitory effect of the gp96 mAb on cell growth was also observed in SKBR3 cells (Figure 4H).

### Targeting gp96 inhibits breast cancer tumor growth

**Figure 5:**
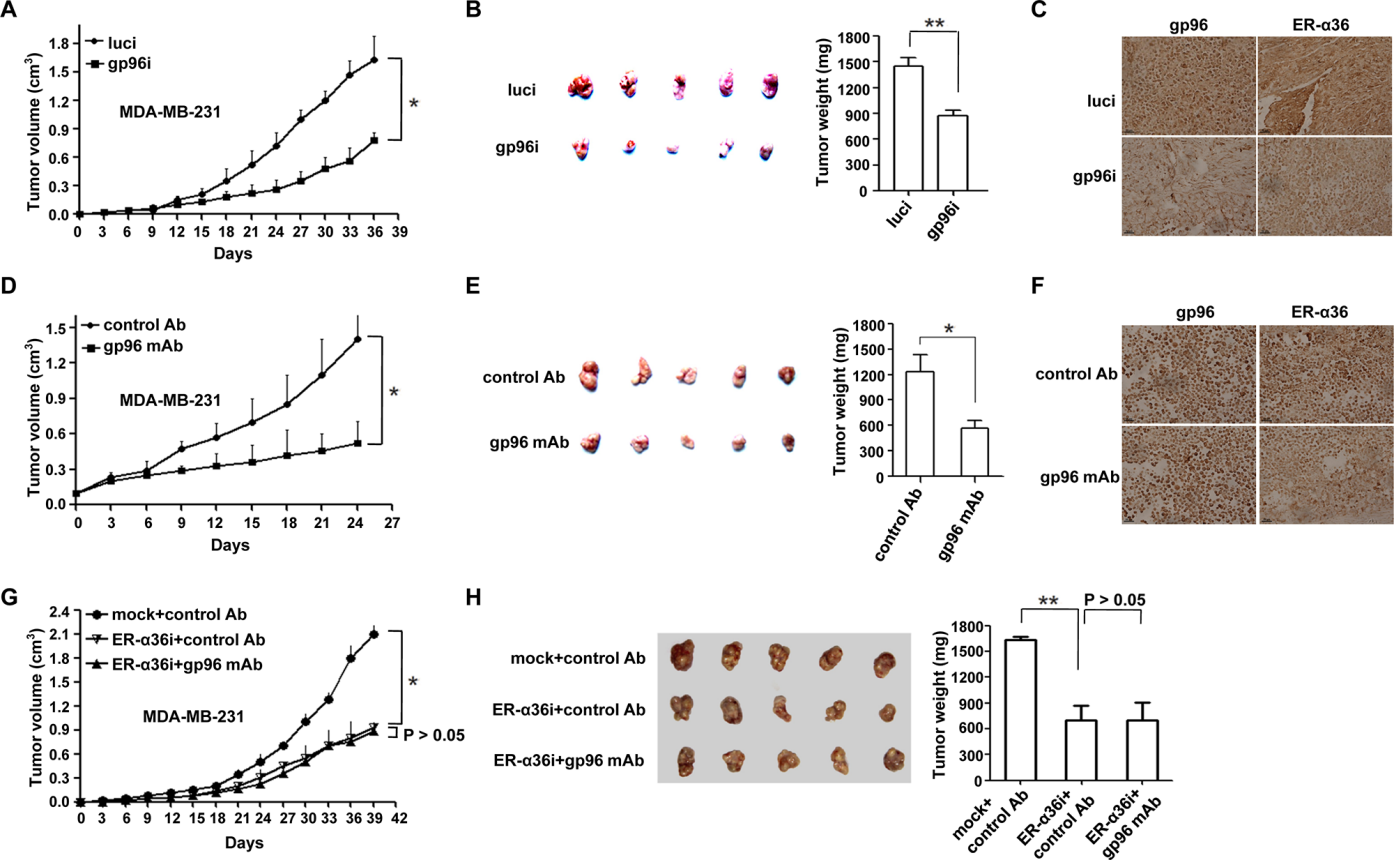
Targeting gp96 with shRNA or mAb leads to suppressed breast tumor growth in mice **A–C.** BALB/c nude mice were injected s.c. with MDA-MB-231-gp96i or MDA-MB-231-luci (mock) cells (model 1). Tumor volume was monitored every 3 days (A) Representative image of tumor growth (left) and mean tumor weights (right) 36 days after cell injection (B) IHC analysis of gp96 and ER-α36 expression in tumors (C) Scale bar, 50 μm. **D–F.** BALB/c nude mice were injected s.c. with MDA-MB-231 cells and treated with the gp96 mAb or control antibody (2mg/kg) when tumors reached a volume of ∼100 mm^3^ (around 2 weeks after injection of tumor cells) (model 2). Tumor volume was monitored every 3 days (D) Representative image of tumor growth (left) and mean tumor weights (right) 3 weeks after gp96 mAb treatment (E) IHC analysis of gp96 and ER-α36 expression in tumors (F) Scale bar, 50 μm. **G.** and **H.** BALB/c nude mice were injected s.c. with MDA-MB-231-ER-α36i or MDA-MB-231-mock cells, and treated with gp96 mAb or control antibody (2mg/kg) (model 3). Tumor volume was monitored every 3 days (E) Representative image of tumor growth (left) and mean tumor weights (right) 39 days after cell injection (F) *P < 0.05, **P < 0.01.

To determine whether gp96 targeting could be an effective strategy to inhibit breast tumor growth *in vivo*, we generated a stable gp96-knockdown cell line, MDA-MB-231-gp96i. Similar to our *in vitro* results, tumor growth was significantly slowed in MDA-MB-231-gp96i xenograft nude mice compared to mock (*P* < 0.05) (Figure 5A). Gp96 depletion resulted in a 39.7% decrease in tumor weights (*P* < 0.01) (Figure 5B). Gp96 knockdown in tumors also decreased ER-α36 expression compared to mock (Figure 5C).

We next determined the therapeutic effect of the gp96 mAb in MDA-MB-231 xenograft nude mice. As shown in Figure 5D and 5E, treatment with the gp96 mAb dramatically slowed tumor growth (*P* < 0.05) and decreased tumor burden by 51.7% (*P* < 0.05) compared to mice treated with control antibody. Treatment with gp96 mAb also decreased ER-α36 levels in xenograft tumors (Figure 5F).

Of note, the inhibitory effect of the gp96 mAb on tumor growth was mostly eliminated with simultaneously silencing ER-α36 (*P* > 0.05), indicating that the gp96 mAb suppressed tumor growth through regulation of ER-α36 (Figure 5G, 5H).

## DISCUSSION

ER-α36 overexpression has been observed in breast cancer [22, 24], adenoid cystic carcinoma (ACC), pure apocrine carcinomas (PAC) of breast [25], endometrial cancer [26], gastric cancer [27], and colorectal cancer [28]. ER-α36 expression is found in breast cancer tumors that are either positive or negative for ER, PR, and Her-2, indicating that ER-α36 might be an additional driver in the development and progression of breast cancer [29]. Due to its emerging roles in the regulation of tumorigenesis and cancer progression, ER-α36 therefore serves as a new potential target for therapeutic development against breast cancer. In this study, we found that mgp96 binds to and stabilizes ER-α36 on the cell membrane of breast cancer cells. Moreover, a gp96 mAb that prevents mgp96 binding to ER-α36 decreases ER-α36 signaling and suppresses breast cancer growth and invasion, both *in vitro* and *in vivo*. These results contribute to our understanding of the modulation of ER-α36 activation and validate mgp96 as a potential therapeutic target for ER-α36-positive breast cancer.

Since ER-α36 activates membrane-initiated non-genomic signaling pathways (MAPK [22, 30], AKT [6], and PKC [31]), cell membrane ER-α36 is thought to play a dominant role in driving breast cancer growth and development. Our results show that mgp96 binds to cell membrane ER-α36, increasing its stability, perhaps by decreasing ubiquitination, and leading to its up-regulation on the membrane of breast cancer cells.

The specificity of the gp96 mAb was verified in our previous study showing that the gp96 mAb only inhibits growth of mgp96-positive breast cancer cells, and has no effect on mgp96-negative cells [13]. Besides, there was no difference in the weight between gp96 mAb-treated mice and control IgG-treated mice, indicating that gp96 mAb inflicts no potential toxicity on mice. In addition, treatment with the gp96 mAb only inhibited proliferation of MDA-MB-231 cells but not gp96-knockdown MDA-MB-231-gp96i cells (data not shown). More studies are needed to determine off-target effects of the gp96 mAb in order to minimize its potential toxicity and improve efficacy.

A previous study revealed that palmitoylation of ERα-66 is necessary for the localization of that particular ER isoform to the cell membrane [32]. It remains to be determined whether post-translational modifications such as palmitoylation also play a role in ER-α36 targeting to the plasma membrane.

In addition to post-transcriptional regulation, ER-α36 transcription is also regulated by many factors. ER-α36 interacts with HER2 in the cytoplasm and membrane of breast cancer cells [15, 29]. ER-α36 positively regulates HER2 expression, and HER2 signaling activates ER-α36 transcription through an AP1 site in the *ESR1* promoter. This positive feedback drives breast cancer development [15]. Additionally, the positive feedback loop of ER-α36/EGFR promotes malignant growth of ER-negative breast cancer cells [14]. As molecular chaperones, HSP90 and synuclein γ (SNCG) also facilitate the expression of ER-α36 and stimulate ligand-dependent cell growth [33]. Interestingly, a recent study demonstrated that the gp96 expression levels are up-regulated by ER-α36 in gastric carcinoma cells [12]. Thus, it is possible that there also exists a positive feedback loop in the mgp96-ER-α36 interaction.

In addition, we previously showed that mgp96 binds to HER2 and enhances HER2 signaling by facilitating HER2 dimerization in HER2-overexpressed breast cancer [13]. Targeting mgp96 with siRNA or specific mAb inhibits HER2-positive breast cancer growth. This may be due to the lack of the ER-α36 ligand E2β in the experimental system. In addition, according to a previous study, there is a positive cross-regulation between HER2 and ER-α36 [15]. Therefore, it is very likely that targeting mgp96 suppresses the growth of HER2-positive breast cancer through inhibition of both HER2 and ER-α36. We speculate that cross-talk among mgp96, ER-α36 and HER2 forms a positive feedback loop in breast cancer, which may affect tumor growth, metastasis, and drug resistance, but this relationship remains to be examined.

We (and others) have shown that gp96 is able to bind antigenic peptides and cross-present the associated peptides to MHC Class I molecules, activating cytotoxic T cell responses [34, 35]. Meanwhile, cell membrane expression of normally ER-resided gp96 is observed in multiple tumors and involved in promoting malignant growth. Targeting mgp96 with specific antibodies may therefore provide a novel therapeutic approach against cancer. More studies are needed to dissect the precise function of gp96 in the context of anti-tumor immunity and targeted therapy. ER-α36 mediates non-genomic estrogen and anti-estrogen (tamoxifen) signaling and stimulates cell proliferation, which contributes to tamoxifen resistance [3, 5, 7]. Given the dominant role of cell membrane ER-α36 in breast cancer growth and development, our study represents an effort to address the underlying mechanism of elevated cell membrane ER-α36 levels mediated by mgp96 in breast cancer. Moreover, our results provide a potential therapeutic strategy for ER-α36 (+) breast cancer via inhibition of mgp96 activity.

## MATERIALS AND METHODS

### Cells, viruses, antibodies, and reagents

ER-negative breast cancer cell lines MDA-MB-231, SKBR3, BT-474, and T47D were obtained from the American Type Culture Collection (Manassas, VA, USA). Recombinant adenoviruses, ad-mgp96 expressing mgp96, and control adenoviruses ad-pDC312 were created by our lab. The ER-α36-knockdown cell line, MDA-MB-231-ER-α36i, and MDA-MB-231-mock cell line, the ER-α36-negative breast cancer cell line MCF7-10A, ER-α36 antibody, E2β and BSA-E2β were generous gifts from Beijing Shenogen Biomedical Co. Ltd. Gp96 polyclonal antibody and Protein G were purchased from Santa Cruz Biotechnology (Dallas, Texas, USA). The gp96 monoclonal antibody (mAb) was generated in our lab. ERK antibody, Phospho-ERK antibody, p38 antibody, and Phospho-p38 antibody were purchased from Cell Signaling Technology (Danvers, Massachusetts, USA). The remaining antibodies were obtained from Zhongshan Goldenbridge Biotechnology (Beijing, China). Cycloheximide (CHX) and MG132 were from Beyotime Institute of Biotechnology (Shanghai, China). Glutathione Sepharose 4B was from GE Healthcare Life Sciences (Little Chalfont, Buckinghamshire, United Kingdom). The protein cross-linkers DTSSP and BS3 were purchased from Thermo Scientific (Waltham, Massachusetts, USA).

### Western blot

Western blot analysis was performed according to our previous description [16].

### Co-immunoprecipitation (co-IP)

Co-IP was performed as previously [13]. Briefly, 2 μg of the relevant antibody was added to cell lysates overnight at 4°C. Then cell lysates were immunoprecipitated with Protein G Sepharose beads for 4 h at 4°C. Immunoprecipitates were separated by SDS-PAGE for Western blot analysis.

### Subcellular fractionation

Cell membrane proteins were isolated using a ProteoExtract™ Subcellular Proteome Extraction Kit (Calbiochem, Germany) following the manufacturer's instructions.

### GST pull-down

Briefly, 20 μg of GST or GST fusion proteins was incubated with 50μl Glutathione Sepharose 4B for 1 h at 4°C, and then, cell lysates were added and incubated for 4 h at 4°C. The agarose beads were washed with PBS twice and resuspended in loading buffer. After incubation in boiling water for 10 min, the supernatant was subjected to Western blot analysis.

### Confocal laser scanning microscopy (CLSM)

Confocal microscopy was performed on non-permeabilized cells as previously described [17]. Images were obtained on a Leica TCS SP2 confocal laser-scanning microscope (Leica Microsystems, Germany).

### Flow cytometry

Cells were pretreated with 0.5 mM EDTA to facilitate removal of substrate and washed with PBS. After blocked in PBS containing 10% BSA, cells were resuspended in a 100 μl PBS volume containing 10% BSA and serially stained with primary and secondary antibodies on ice for 1 h. Detection of fluorescence intensity was performed on a FAC-Scan cytometry machine (BD Biosciences, USA).

### Cell proliferation

Cell growth was measured using a Cell Counting Kit-8 (CCK-8) (Dojindo, Japan) following the manufacturer's instructions.

### Cell invasion

Cell invasion assays were performed as previously described [16].

### Establishment of a stable shRNA cell line

The short hairpin RNA (shRNA) sequence targeting the gp96 gene was designed and synthesized as previously described [18]. The shRNA construct was established by inserting the oligonucleotides into the RNA interference (RNAi)-pSIREN-RetroQ vector. The recombinant plasmid, pSIREN-gp96i, was confirmed by sequencing. Luciferase shRNA was selected as a mock transfection control (pSIREN-luci). Phoenix cells were co-transfected with pSIREN-gp96i or pSIREN-luci and the helper vector. Seventy-two hours after transfection, the supernatant was collected and MDA-MB-231 cells were infected with the virus suspension. At 48 h after infection, MDA-MB-231 cells were selected with 2 μg/ml puromycin for 2 weeks to establish stable shRNA cell lines: MDA-MB-231-gp96i and MDA-MB-231-luci (mock). The protein levels of gp96 were analyzed by western blotting to confirm that gp96 was effectively silenced.

### Animal experiments

Model 1 – Six-week-old female BALB/c nude mice were randomly divided into two groups (*n* = 5/group). MDA-MB-231-gp96i or MDA-MB-231-luci (mock) cells were maintained in phenol red-free media with 2.5% charcoal-stripped fetal calf serum for three days, and a total of 1 × 10^7^ cells were injected subcutaneously (s.c.) in the right hind flank of nude mice 5 days after s.c. implantation of 1.7 mg/60-day release E2 pellets (Innovative Research of American, Sarasota, FL). Tumor growth was monitored every 3 days and tumor size was calculated with the formula: Tv = (L × W^2^)/2. Mice were sacrificed 36 days after cell injection for tumor weight evaluation and immunohistochemistry (IHC).

Model 2 – MDA-MB-231 cells were maintained in phenol red-free media with 2.5% charcoal-stripped fetal calf serum for three days, and a total of 1 × 10^7^ cells were injected s.c. in the right hind flank of six-week-old female BALB/c nude mice 5 days after s.c. implantation of 1.7 mg/60-day release E2 pellets. Tumor growth was monitored every 3 days. Mice were randomly divided to two groups (*n* = 5/group) when tumors reached a volume of ∼100 mm^3^ (around 2 weeks after injection of MDA-MB-231 cells). Mice were treated with the gp96 mAb or control antibody (2 mg/kg) via intraperitoneal (i.p.) injection twice a week. Three weeks later, mice were sacrificed for tumor weight evaluation and IHC.

Model 3 – Six-week-old female BALB/c nude mice were implanted s.c. with 1.7 mg/60-day release E2 pellets 5 days before cell injection and randomly divided into three groups (*n* = 5/group), and MDA-MB-231-mock and MDA-MB-231-ER-α36i cells were maintained in phenol red-free media with 2.5% charcoal-stripped fetal calf serum for three days: group 1 (231-mock+control Ab) was injected s.c. in the right hind flank with 1 × 10^7^ MDA-MB-231-mock cells, and treated with control antibody (2 mg/kg) via i.p. injection twice a week from the day 15 after cell injection; group 2 (231-ER-α36i+control Ab) was injected s.c. in the right hind flank with 1 × 10^7^ MDA-MB-231-ER-α36i cells, and treated with control antibody (2 mg/kg) via i.p. injection twice a week from the day 15 after cell injection; group 3 (231-ER-α36i+gp96 mAb) was injected s.c. in the right hind flank with 1 × 10^7^ MDA-MB-231-ER-α36i cells, and treated with gp96 mAb (2 mg/kg) via i.p. injection twice a week from the day 15 after cell injection. Tumor growth was monitored every 3 days. All mice were sacrificed at the day 39 after cell injection for tumor weight evaluation.

Mice were maintained and cared for in strict compliance with the institution's guidelines of the Institute of Microbiology, Chinese Academy of Sciences of Research Ethics Committee. All procedures were approved by the Research Ethics Committee.

### Immunohistochemistry (IHC)

IHC analysis of paraffin-embedded mouse tumors was performed as described previously [18].

### Statistical analysis

All data are presented as the mean ± SD, and significance was determined by two-tailed Student's *t* test. A *P* value of less than 0.05 was considered statistically significant.
